# Analysis of Blood Transfusion Data Using Bivariate Zero-Inflated Poisson Model: A Bayesian Approach

**DOI:** 10.1155/2016/7878325

**Published:** 2016-09-14

**Authors:** Tayeb Mohammadi, Soleiman Kheiri, Morteza Sedehi

**Affiliations:** ^1^Student's Research Committee, Shahrekord University of Medical Sciences, Shahrekord, Iran; ^2^Social Health Determinants Research Center, Shahrekord University of Medical Sciences, Shahrekord, Iran; ^3^Epidemiology and Biostatistics Department, Shahrekord University of Medical Sciences, Shahrekord, Iran

## Abstract

Recognizing the factors affecting the number of blood donation and blood deferral has a major impact on blood transfusion. There is a positive correlation between the variables “number of blood donation” and “number of blood deferral”: as the number of return for donation increases, so does the number of blood deferral. On the other hand, due to the fact that many donors never return to donate, there is an extra zero frequency for both of the above-mentioned variables. In this study, in order to apply the correlation and to explain the frequency of the excessive zero, the bivariate zero-inflated Poisson regression model was used for joint modeling of the number of blood donation and number of blood deferral. The data was analyzed using the Bayesian approach applying noninformative priors at the presence and absence of covariates. Estimating the parameters of the model, that is, correlation, zero-inflation parameter, and regression coefficients, was done through MCMC simulation. Eventually double-Poisson model, bivariate Poisson model, and bivariate zero-inflated Poisson model were fitted on the data and were compared using the deviance information criteria (DIC). The results showed that the bivariate zero-inflated Poisson regression model fitted the data better than the other models.

## 1. Introduction

Blood transfusion is so important in health system that it plays a big part in saving many people's lives in normal and emergency situations. Furthermore, it has a noticeable impact on improving the quality of life and consequently the life expectancy of chronic patients. Nevertheless, many patients either die due to lack of access to safe blood transfusion or at least suffer from it. According to the World Health Organization report, about one percent of the population of every country is in need of blood donation [1]. Today, the need for blood and its products is increasing day by day [2]. Since some diseases may be caused by blood transfusion, screening the donors and detecting potential healthy donors are of great importance [3]. For that matter, lack of healthy donors has always been a serious problem for blood banks to supply sufficient and healthful blood [4, 5]. Therefore, one of the main goals of blood transfusion centers is detecting and preserving healthy donors and preventing unhealthful blood donation which may cause many diseases to be created or aggravated [2]. Nonetheless, even from among those who are eligible to donate blood only a small portion really become blood donors [6]. Inasmuch as the screening test is done at each donation to separate healthful from unhealthful blood, the more the blood donation is, the higher the chances of getting healthful blood will be. That is why recognizing the factors which influence blood donation is of great importance in attracting potential donors and turning them into regular donors [3]. From among all the laboratory screening methods to prevent the transference of infection through blood, the only truly effective method is to select healthy donors and not allow ineligible donors to donate blood [7]. People who are not eligible to donate blood are called “deferred donors” [8]. Most deferrals are “temporary” and exist due to taking certain medications before donation, high or low blood pressure, anemia, high-risk behavior, and so forth. These deferrals are controllable and can be reduced by giving the necessary information before donation [2, 9–11]. Naturally, as return to donation increases, the probability of deferral from donation increases too. Therefore, it is expected that there will be positive correlation between the number of donation and the number of deferral. What has been said so far points out the importance of getting healthful blood and its products and the relation between number of return for donation and deferral.

Poisson regression model belongs to the family of generalized linear models in which the response variable is a count one and has followed Poisson distribution. The equality of the variance and the mean of the dependent variable is one of the important hypotheses of Poisson regression analysis. In most practical applications, response observations are overdispersed (i.e., the variance of observations is significantly bigger than their mean); thus fitting the Poisson regression model on the data will not yield the desired results. In a univariate case, the best solution to the problem of overdispersion is to use a negative binomial regression model [12, 13]. In many studies, it is seen that there is a correlation between the two count response variables; in these cases, dealing with the response variables separately without considering this correlation will result in inconsistent and inefficient estimators [14]. The basic solution is to use bivariate count models [14–16]. In medical, environmental, and ecological studies, existence of excessive zeros in count data is common. If the zeros are ignored for the sake of simplifying the analysis, valuable information will be lost and can result in biased estimate of the parameters and thus misleading findings [17, 18]. An appropriate class to explain these data is the class of zero-inflated distributions. In practice, the data including zero inflation can be sampled from zero-inflated Poisson or zero-inflated negative binomial [18]. The zeros in the count data can then be attributed to structural causes (known as structural zeros) or sampling limitations (known as sampling zeros) [17]. The most common model for explanation and analysis of excessive zeros in count data is the zero-inflated model [17, 18]. Generally, for this kind of data, zero-inflated models fit better than regular models [19]. For a multivariate and especially a bivariate case, in which there is a correlation between the two count response variables [18], since the marginal distributions of bivariate model are univariate, this bivariate model cannot be used to model extra zeros paired count data. Instead, the bivariate zero-inflated regression model is used [20]. In most applications, it seems logical to use the zero-inflated bivariate Poisson distribution.

Due to calculation problems while fitting these models, researchers were not able to use zero-inflated bivariate count model for a long time [18]. Recent improvements in hierarchical Bayesian modeling and specifically the improvement in simulation methods like Markov chain Monte Carlo (MCMC) have provided the mechanisms for simple implementation of bivariate distributions such as bivariate Poisson [18].

This study seeks to determine the factors which affect the number of return for donation and also deferral from donation. The data is of the type of count data and, to explain it, it is necessary to use a count regression model. On the other hand, the data has a large number of zeros, and also there is a positive correlation between the number of return for donation and the number of deferral. In order to model these two variables obtained from blood transfusion data, the bivariate zero-inflated Poisson regression model was used. The remainder of the paper is organized as follows: in Section 2, first, the bivariate data set of blood transfusion is introduced, then, the bivariate zero-inflated Poisson models are presented, and later a Bayesian methodology for fitting the bivariate zero-inflated Poisson model is developed. In Section 3, the result of fitting the proposed model on blood transfusion data is brought up and discussed. Finally, Section 4 provides some conclusions.

## 2. Materials and Methods

### 2.1. Data

**Figure 1:**
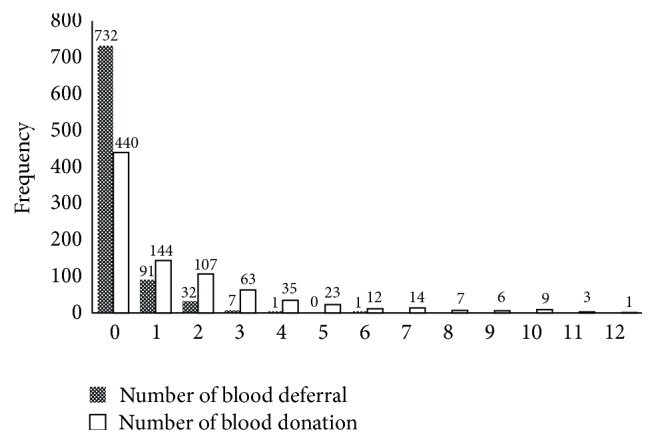
Frequency of the response variables.

The data used in this research was obtained from a longitudinal study in which a random sample of donors who had a first-time successful donation were followed up for a maximum of five years and their number of return for blood donation and number of blood deferral were measured as response. A full description of the data can be seen in [3].  Figure 1 shows the frequency of return for blood donation and blood deferral. 51% of return for blood donation and 85% of blood deferral are zero, which is much more than the Poisson distribution contribution. On the other hand, the Spearman correlation coefficient of the number of return for donation and the number of deferral was equal to 0.276, which is significant at level 0.01. Therefore, to study the effective factors, the bivariate zero-inflated regression model was used. Sex, weight, age, marital status, education, and job were taken as independent variables. Since education and job were nominal, in order to apply those in the model, three dummy variables for education and four for job were used. As a result, thirteen independent variables were inserted into the model.

### 2.2. Statistical Models

#### 2.2.1. Bivariate Poisson Distribution

The discrete random variables of *Y*_1_ and *Y*_2_ are bivariate Poisson distribution and are shown as (*Y*_1_, *Y*_2_) ~ BP(*λ*_1_, *λ*_2_, *λ*_3_) if their joint probability mass is given by

(1) fBPy1,y2=PY1,Y2y1,y2=PY1=y1,Y2=y2=e−λ1+λ2+λ3λ1y1y1!λ2y2y2!·∑k=0min⁡⁡y1,y2y1ky2kk!λ3λ1λ2k,

where *λ*_*i*_ > 0 and *y*_1_, *y*_2_ = 0, 1, 2, 3,…. Marginal distribution of *Y*_1_ and *Y*_2_ is Poisson distribution with parameters of *λ*_1_ + *λ*_3_ and *λ*_2_ + *λ*_3_, respectively. Mean and variance of *Y*_1_ and *Y*_2_ are *λ*_1_ + *λ*_3_ and *λ*_2_ + *λ*_3_, respectively. The covariance between *Y*_1_ and *Y*_2_ is *λ*_3_, which shows a measure of dependency between *Y*_1_ and *Y*_2_. Bivariate Poisson distribution can be defined using latent variables. Assuming *Z*_1_, *Z*_2_, and *Z*_3_ are mutually independent Poisson random variables with parameters *λ*_1_, *λ*_2_, and *λ*_3_, respectively, then the covariance between *Y*_1_ and *Y*_2_ will be

(2) $$\begin{array}{c} \text{Cov}\left(\;Y_{1},Y_{2}\;\right)=\text{Var}\left(\;Z_{3}\;\right)=\lambda_{3} \end{array}$$

and the coefficient of correlation between *Y*_1_ and *Y*_2_ is given as

(3) CorrY1,Y2CovY1,Y2VarY1VarY2=λ3λ1+λ3λ2+λ3

if *λ*_3_ = 0; we have a special case which yields that *Y*_1_ and *Y*_2_ are independent. This way, the bivariate Poisson distribution becomes multiplication of two univariate Poisson distributions and is known as double-Poisson model [16, 21].

#### 2.2.2. Bivariate Zero-Inflated Poisson Distribution

Here, we use a bivariate zero-inflated Poisson (BZIP) model, which is a mixture of bivariate Poisson and a point mass at (0,0). The joint probability mass function of BZIP model is defined as [22, 23]

(4) fBZIPy1,y2=PY1=y1,Y2=y2=P0+1−P0exp⁡−λ,y1=y2=01−P0fBPy1,y2 ∣ λ1,λ2,λ3,y1≠0  or  y2≠0,

where 0 < *P*_0_ < 1 and *λ* = *λ*_1_ + *λ*_2_ + *λ*_3_.

The marginal distributions of BZIP model are zero-inflated univariate Poisson distribution, whose probability mass function is

(5) PYk=yk=P0+1−P0exp⁡−λk−λ3,yk=01−P0λk+λ3ykyk!exp⁡−λk−λ3,yk≠0k=1,2.

The moments of bivariate zero-inflated Poisson distribution are given as [23]

(6) EYk=1−P0λk+λ3,VarYk=EYk1+P0λk+λ3,k=1,2,EY1Y2=1−P0λ1+λ3λ2+λ3+λ3,CovY1,Y2=1−P0λ3+P0λ1+λ3λ2+λ3.

### 2.3. Bayesian Estimation

Assuming (*y*_*i*1_, *y*_*i*2_), *i* = 1,…, *n*, is an observation from the bivariate zero-inflated Poisson and ${\underline{x}}_{ki},\;\;k=1,2,3$, is the vector of explanatory variables, in the zero-inflated Poisson regression model, an exponential link function is used to relate the explanatory variables to the *λ*_*k*_, *k* = 1,2, 3 [15, 24].

(7) log⁡λ1i=x_1iTβ,log⁡λ2i=x_2iTγ,log⁡λ3i=x_3iTα.

It is also possible to link explanatory variables to mass index (*P*_0_) via logit link function. We assume **β** and **γ** are vectors of regression parameters for *λ*_1_ and *λ*_2_, respectively, and let *λ*_3_ = exp⁡(*α*).

Suppose *θ* = (**β**, **γ**, **α**) is a vector of all regression parameters. We choose noninformative prior distributions for regression parameters as normal with zero mean and large variance of 1000. *β*_*i*_ ~ *N*(0, 1000), *γ*_*i*_ ~ *N*(0, 1000),  *i* = 1, …, *p*, and *α* ~ *N*(0, 1000).

Also, we choose uniform distribution in the (0,1) interval for *P*_0_. We assume that the prior distributions of all parameters of model are independent of each other. Suppose there are *n* individuals under study whose responses are represented by *y*_*i*_ = (*y*_*i*1_, *y*_*i*2_)′; *i* = 1, …, *n* and *D*_obs_ = (*y*_1_,…, *y*_*n*_) is a vector of all observations. If *L*(*D*_obs_∣*θ*, *P*_0_) is likelihood function and *π*(*θ*) and *π*(*P*_0_) are prior distributions of *θ* and *P*_0_, then the joint distribution of the data and the parameters is

(8) $$\begin{array}{c} \pi \left(\;D_{obs},\theta ,P_{0}\;\right)=L\left(\;D_{obs}\;|\;\theta ,P_{0}\;\right)\cdot \pi \left(\;\theta \;\right)\cdot \pi \left(\;P_{0}\;\right). \end{array}$$

The posterior distribution of (*θ*, *P*_0_) will be
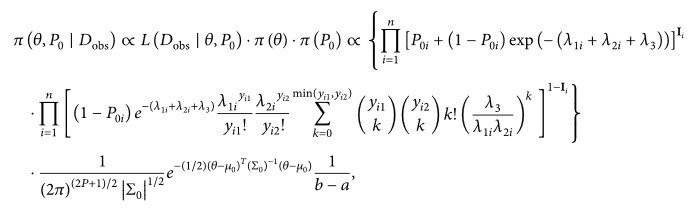
(9)in which **I**_*i*_ is an indicator that (*Y*_1_, *Y*_2_) are both zero at which **I**_*i*_ = 1 and 0 otherwise. The posterior density of parameters is very complicated; thus, we use simulation approach of Markov chain Monte Carlo (MCMC) to estimate the parameters.

## 3. Results and Discussion

The codes of the programs related to the models were written using the WinBUGS software version 1.4.3. To make sure of the convergence of parameter's chains, both graphical and analytical convergence diagnostics were used. In the graphical method, the behavior of the trace plots and the autocorrelation function were checked. Also, in order to study the convergence analytically, two parallel chains with a burn-in of 5000 samples and 30000 observations after that were run. The samples obtained from posterior distributions of the parameters were fed into R software and the modified Gelman-Rubin [25] scale reduction factor of the model's parameters was calculated using package of BOA [26]. All these values were close to one; the biggest value of scale reduction factor was 1.00402, which belongs to the coefficient of sex. We found no evidence from the convergence diagnostics methods that the chains are not converging to posterior distribution of the parameters. Since there was a high autocorrelation in the successive values of the simulated observation, in order to get roughly independent samples, every 20th sample was kept to get statistical summaries of the parameters. The posterior summaries of the parameters of the bivariate zero-inflated Poisson regression model, including mean, median, standard deviation, and 95% credible intervals, based on 30,000 simulated values in presence and absence of independent variables can be seen in Tables 1 and 2, respectively. In order to prevent confusion in the tables, the MC error values have not been reported, yet they were taken lower than 0.1 of the posterior standard deviation for all parameters, which in turn indicates the convergence of the produced chains [27].

The posterior mean and 95% credible interval for the zero-inflation parameter (proportion of extra zeros) in model (*P*_0_) are 0.43 and (0.391,0.466), respectively. In other words, in BZIP regression model, the posterior estimate of the parameter *P*_0_ = *P*(*Y*_1_ = 0, *Y*_2_ = 0) is equal to 0.43. Since the number of the observed (0,0) in the response variables was 411, the observed proportion of zeros was 411 to 864, that is, 0.48. This value is close to the posterior estimate of the inflation parameter obtained from the BZIP regression model. In the univariate model, the posterior estimate of extra zero parameters in the zero-inflated Poisson regression model is obtained at 0.448 for the number of return for donation and 0.67 for the numbers of blood deferral. So, if the dependency between the two response variables is not considered, and the responses of the number of return for donation and deferral are assumed independent, then the probability of *P*(*Y*_1_ = 0, *Y*_2_ = 0) will be equal to *P*(*Y*_1_ = 0) · *P*(*Y*_2_ = 0), which will be estimated as 0.448*∗*0.67, and result is 0.30, which is an underestimation compared to the BZIP model and the true proportion of zeros. The estimated value of the correlation coefficient of the BZIP model in absence of covariate using estimated moments is equal to 0.282, which is very close to the Spearman correlation coefficient of the original data (*r* = 0.276) that, in turn, shows the qualified fitting of the model.

The 95% credible intervals of regression parameters showed that weight and job (student and unemployed versus housekeeper) were important factors to the number of blood donation. Results, also, showed that weight is an important factor to the number of blood deferral. To compare the models, deviance information criteria (DIC) were used [28]; the results of DIC are presented in Table 3.

The findings showed that, among the independent variables, the only ones which influenced the number of donation were weight and job (as the weight increases, return for donation increases too). Also, students returned to donate, most of all jobs, and housekeepers did so the least of all. Weight had a positive effect on the number of blood deferral as well; it looks like people with higher weights return for donation more than other people, which in turn causes a higher rate of blood deferral too.

All the prior distributions for parameters of this model were noninformative, so the values of hyperparameters of prior distributions were selected in a way that their variance would be bigger. As another study, it is proposed to use bivariate zero-inflated negative binomial model with our data and results could be compared with BZIP model.

## 4. Conclusion

This article mainly aims at discovering the factors influencing number of return for blood donation and number of blood deferral. To do so, the bivariate zero-inflated Poisson regression model was applied using a Bayesian approach via MCMC simulation. Since the proposed response is naturally bivariate, using ordinary univariate zero-inflated regression models will not yield the desired results. A positive correlation between dependent variables and zero frequency more than Poisson distribution in each of the dependent variables are the main characteristics of the data in this study. The idea of using the bivariate zero-inflated Poisson (BZIP) model was strengthened by these two features because this model not only explains the positive correlation between the two responses (as BP model does) but also explains overdispersion and underdispersion conditions (which BP does not). The bivariate zero-inflated Poisson model is an ideal model as it has overcome the problems of bivariate Poisson model [15].

According to the estimate of the DIC in Table 3, the BZIP model fits better and more comprehensively on the data in comparison with the bivariate Poisson model. Moreover, the total DIC value for the double-Poisson model (two individual univariate Poisson models) equals the sum of DIC of each model [19] and will be 4413.91, which is significantly lower than the DIC statistic 4420.72 from the BP model. This finding indicates that despite considering the correlation between the responses, the BP model cannot fit the data very well. On the other hand, the total DIC value for two individual univariate ZIP models is equal to the sum of DIC statistic of each model and will be 3866.8, while this value was 3852.23 for BZIP model which shows its better fitting. This provides empirical support for the BZIP model over univariate ZIP models for our data. Among univariate Poisson models, for each of the response variables, the DIC value for the zero-inflated model was lower than that of the ordinary model, which is logical due to the existence of excessive zeros in both responses. Out of the models fitted in this study, the BP model had the most complexity. Overall, the bivariate zero-inflated Poisson regression model was the most comprehensive and the best model to explain the correlation and excessive zeros and fitted the blood transfusion data in this study better than other models.

## Figures and Tables

**Table 1:** Posterior summaries for the parameters of bivariate zero-inflated Poisson model.

Parameter	Mean	Median	Standard deviation	95% credible interval	
				2.5 percentile	97.5 percentile
*λ*_1_	2.541	2.540	0.079	2.390	2.697
*λ*_2_	0.388	0.388	0.029	0.334	0.447
*λ*_3_	1.732*E* − 4	1.996*E* − 11	9.551*E* − 4	3.941*E* − 32	0.002

*P*_0_	0.446	0.446	0.018	0.412	0.482
Deviance	3907	3906	3.627	3903	3916

**Table 2:** Posterior summaries for the parameters of bivariate zero-inflated Poisson regression model.

Parameter	*λ*_1_					*λ*_2_				
	Mean	Median	Standard deviation	2.5 percentile	97.5 percentile	Mean	Median	Standard deviation	2.5 percentile	97.5 percentile
Beta 0 (constant)	−0.0143	−0.011	0.250	−0.515	0.460	−0.780	−0.773	0.403	−1.6	0.005
Beta (age − mean (age))	−0.004	−0.004	0.004	−0.012	0.004	0.0041	0.0042	0.01	−0.015	0.023
Beta (sex)	0.43	0.421	0.28	−0.01	0.99	−0.414	−0.429	0.5	−1.314	0.601
Beta (weight − mean (weight))	0.0161	0.0161	0.0027	0.011	0.0213	0.0158	0.0159	0.0064	0.0032	0.0284
Beta (marital status)	0.03	0.03	0.088	−0.141	0.202	0.176	0.177	0.21	−0.239	0.59

Education										
Beta (high school versus elementary)	0.0963	0.0964	0.11	−0.111	0.304	0.358	0.357	0.271	−0.171	0.892
Beta (diploma versus elementary)	−0.07	−0.07	0.11	−0.28	0.148	0.39	0.39	0.271	−0.127	0.93
Beta (university versus elementary)	−0.25	−0.25	0.13	−0.5	0.002	0.241	0.238	0.33	−0.41	0.9

Job										
Beta (clerical versus housekeeper)	0.57	0.57	0.336	−0.1	1.23	−0.707	−0.702	0.575	−1.9	0.396
Beta (worker versus housekeeper)	0.533	0.532	0.332	−0.12	1.2	−0.462	−0.455	0.566	−1.6	0.63
Beta (free job versus housekeeper)	0.35	0.35	0.33	−0.3	1.01	−0.3	−0.259	0.553	−1.4	0.77
Beta (student versus housekeeper)	0.81	0.812	0.34	0.15	1.5	−0.48	−0.473	0.6	−1.67	0.65

*P*_0_	0.428	0.429	0.020	0.391	0.466					
*λ*_3_	2.075*E* − 4	2.206*E* − 11	0.0012	2.596*E* − 32	0.0023					
Deviance	3827	3826	7.063	3815	3844					

**Table 3:** Results of deviance information criterion.

Model	*P*_*D*_	$\overset{-}{Dbar}$	DIC
Poisson (*Y*_1_)	11.93	3373.47	3385.4
Poisson (*Y*_2_)	11.81	1016.70	1028.51
Zero-inflated Poisson (*Y*_1_)	13.01	2885.16	2898.2
Zero-inflated Poisson (*Y*_2_)	12.90	955.69	968.60
Bivariate Poisson (*Y*_1_, *Y*_2_)	27.10	4393.64	4420.72
Bivariate zero-inflated Poisson	25.32	3826.91	3852.23
